# Aesthetic lip filler augmentation is not free of adverse reactions: lack of evidence-based practice from a systematic review

**DOI:** 10.3389/froh.2024.1495012

**Published:** 2024-10-17

**Authors:** M. Coppini, V. C. A. Caponio, R. Mauceri, G. Pizzo, N. Mauceri, L. Lo Muzio, G. Campisi

**Affiliations:** ^1^ Department of Precision Medicine in Medical, Surgical and Critical Care, University of Palermo, Palermo, Italy; ^2^ Department of Biomedical and Dental Sciences and Morphofunctional Imaging, University of Messina, Messina, Italy; ^3^ Department of Clinical and Experimental Medicine, University of Foggia, Foggia, Italy; ^4^ Unit of Oral Medicine and Dentistry for fragile Patients, Department of Rehabilitation, Fragility and Continuity of Care University Hospital Palermo, Palermo, Italy; ^5^ Department of Biomedicine, Neuroscience and Advanced Diagnostics (Bi.N.D), University of Palermo, Palermo, Italy

**Keywords:** dermal fillers, hyaluronic acid, lip filler, drug-related side effects and adverse reactions, systematic review

## Abstract

**Introduction:**

In the last decades, dermal fillers have gained widespread acceptance for cosmetic purposes since their approval for different health conditions, including lip augmentation and aesthetic intervention of the face. Unfortunately, while filler lip procedures are performed using biomaterials with improved physical characteristics, they are not devoid of adverse drug reactions (ADRs), including those with late-onset.

**Methods:**

This systematic aims to investigate the ADRs associated with lip augmentation procedures using dermal fillers. A systematic review search was conducted in Medline/PubMed, Scopus, Web of Science to answer the PEO question: What are the ADRs in patients undergoing lip augmentation procedures with dermal fillers, and how frequent are they?

**Results:**

The risk of bias was assessed, and a systematic review was conducted. Nineteen studies were included. In total, 30 patients affected by filler lip ADRs were analyzed, of which 29 were females and only 1 was male with a mean age of 50.9 ± 12.8 years. Hyaluronic acid was the most commonly dermal filler used and granulomatous foreign body reaction was the most common filler lip reaction reported. The mean time between filler lip injection and granulomatous foreign body reaction onset was 57.9 ± 54 months (median 24 months).

**Discussion:**

No study reported ADRs to regulatory authorities. Our results indicate that adverse reactions can occur even long-term after the aesthetic procedure. Therefore, ongoing short-term and long-term follow-up visits are essential, as biocompatible materials are not free from ADRs. Additionally, a lack of reporting ADRs to regulatory authorities has emerged, which is crucial for patient safety.

**Systematic Review Registration:**

https://www.crd.york.ac.uk/prospero/display_record.php?RecordID=534656, identifier: CRD42024534656.

## 1 Background

The concept of adverse drug reaction (ADR) was introduced in 1972 by the World Health Organization to indicate “a response to a drug that is harmful, unintended and occurs at doses normally used by humans for the prevention, diagnosis, or therapy of a disease, or to modify its physiological functions" (1). The definition was upgraded over time, and the last definition was provided by the Directive 2010/84/EU, describing ADR as “a response to a medicinal product which is noxious and unintended”. The new definition also includes ADR observed with non-authorized uses of the drug, specifically off-label use, medical errors, misuse, abuse, and occupational exposure (2). According to Aronson and Edwards classification, six types of ADR are generally observed (augmented, bizarre, chronic, delayed, end of use, and failure) (3).

The constant introduction of new active molecules on the drug market, changes in clinical indications, and potential drug interactions require careful monitoring of ADRs. This is the task of pharmacovigilance, defined as “the science and activities related to the identification, evaluation, understanding and prevention of ADRs" (4). Timely identification and reporting of ADRs are essential for patients’ safety and regulatory decision-making.

Although dermal fillers are not precisely drugs, they have been categorized as “medical devices” by the U.S. Food and Drug Administration (FDA). Also in Europe, according to the Medical Devices Regulation, dermal fillers are classified as class III medical devices, the highest risk class, if their ingredients are absorbed, as is the case with hyaluronic acid and collagen (5). In the last decades, dermal fillers have gained widespread acceptance for cosmetic purposes since they were approved by the FDA for different indications, including augmentation of lips, cheeks and chin. The International Society of Plastic Surgery (ISPS) put out global data showing 3.7 million hyaluronic acid filler procedures in 2018, making it the second most often performed procedure in the world, after botulinum toxin (6).

The aesthetic treatment with injectable dermal fillers is considered a relatively safe minimally invasive procedure, but as with any medical procedure, there are risks involved with their use (7). Side effects may be related to the filler material itself (e.g., a non-FDA-approved dermal filler injection), or other factors, including injection technique and patients’ immune responses (8).

Throughout the world, approximately 160 different injectable fillers are currently available on the market. These different products can be distinguished in terms of their duration, the risk profile, the injection depth (dermal, subcutaneous, supraperiosteal) as well as the origin of the filler substance (human or cadaveric-derived, animal, bacterial fermentation, or synthesis) (9, 10). The duration of dermal fillers depends on the absorption time of the same.

Depending on the absorption time, dermal fillers are classified as temporary (e.g., hyaluronic acid and collagen), semipermanent (e.g., Poly-L-lactic acid, Polymethyl methacrylate), or permanent (e.g., silicone) (11). Due to its biological properties and reversibility using the hyaluronidase enzyme, hyaluronic acid (HA) has become the most widely used filler material for lip augmentation (12). In fact, in cases of overcorrection or complications, HA can be effectively dissolved with hyaluronidase, offering a level of safety and control that is not possible with other dermal fillers, which are not easily corrected once injected (13).

Unfortunately, although filler lip procedures are performed using biomaterials with improved physical characteristics, they are not devoid of early and delayed ADR.

To reduce severe outcomes, readmissions to hospitals, overall hospital expenses, and future ADR incidences and improve patients’ quality of life, it is mandatory to report ADRs to the regulatory agency, when they occur (14). Often, these conditions are not highlighted or reported to the appropriate authorities, resulting in the underreporting of oral ADRs (15).

A recent study conducted by Al Mashhrawi YM et al. reported that more than half of patients who undergo cosmetic fillers, including filler lips, were unaware of the complications they could cause. Furthermore, according to this study, the main source of information on fillers was social media, followed by the Internet, and only in 17.1% of cases patients were informed by physicians (16).

The present systematic review was performed to investigate the ADRs associated with lip augmentation procedures using dermal fillers.

## 2 Material and methods

### 2.1 Protocol

A systematic literature search was conducted independently by two authors (MC and VCAC). The protocol for this study was designed following the Preferred Reporting Items for Systematic Reviews and Meta-Analyses (PRISMA) guidelines (17). Prospectively, the protocol for this systematic review has been registered on the International prospective register of systematic reviews (PROSPERO) with the following registration code: CRD42024534656.

### 2.2 PIO and research question

The research question was designed based on PIO items in which:

P: Patients referring to the medical attention for aesthetic improvements of the lips

I: Lip augmentation procedures with dermal filler

O: Adverse Drug Reaction

The systematic review was based on the following research question: “What are the oral adverse reactions in patients undergoing lip augmentation procedures with dermal fillers, and how frequent are they?”

### 2.3 Data sources and search strategy

A selection of studies concerning oral adverse reactions associated with filler lips was performed by two authors (MC and VCAC). Records were identified using different search engines (e.g., Medline/PubMed, Scopus, Web of Science) and by scanning reference lists of articles. For the search strategy, MeSH terms and free text words were combined through Boolean operators as follows: (“Drug-Related Side Effects and Adverse Reactions” OR “Injection Site Reaction” OR “Drug Hypersensitivity” OR ADR OR “side effect” OR “adverse reaction” OR “adverse effect”) AND (lip OR mouth OR “Lip injection”) AND (“Dermal Filler” OR Filler OR “Hyaluronic Acid” OR Botulinum) NOT (“systematic review” OR “meta-analysis”). The research was completed in March 2024.

### 2.4 Eligibility criteria

The inclusion criteria for the studies were as follows:

• Human studies independently of the study design, such as case reports, series, case-control, cohort and randomized clinical trials.
• English language
• Only patients that underwent lip augmentation with dermal fillers
• Studies that included specific patient case information, reporting ADRs to the specific intervention.

Exclusion criteria were studies focused on different types of anatomical filler delivering (e.g., nasolabial fold, glabella, chin or perioral area), studies with missing patient information, narrative and systematic reviews and meta-analyses.

### 2.5 Study selection and data collection process

**Figure 1:**
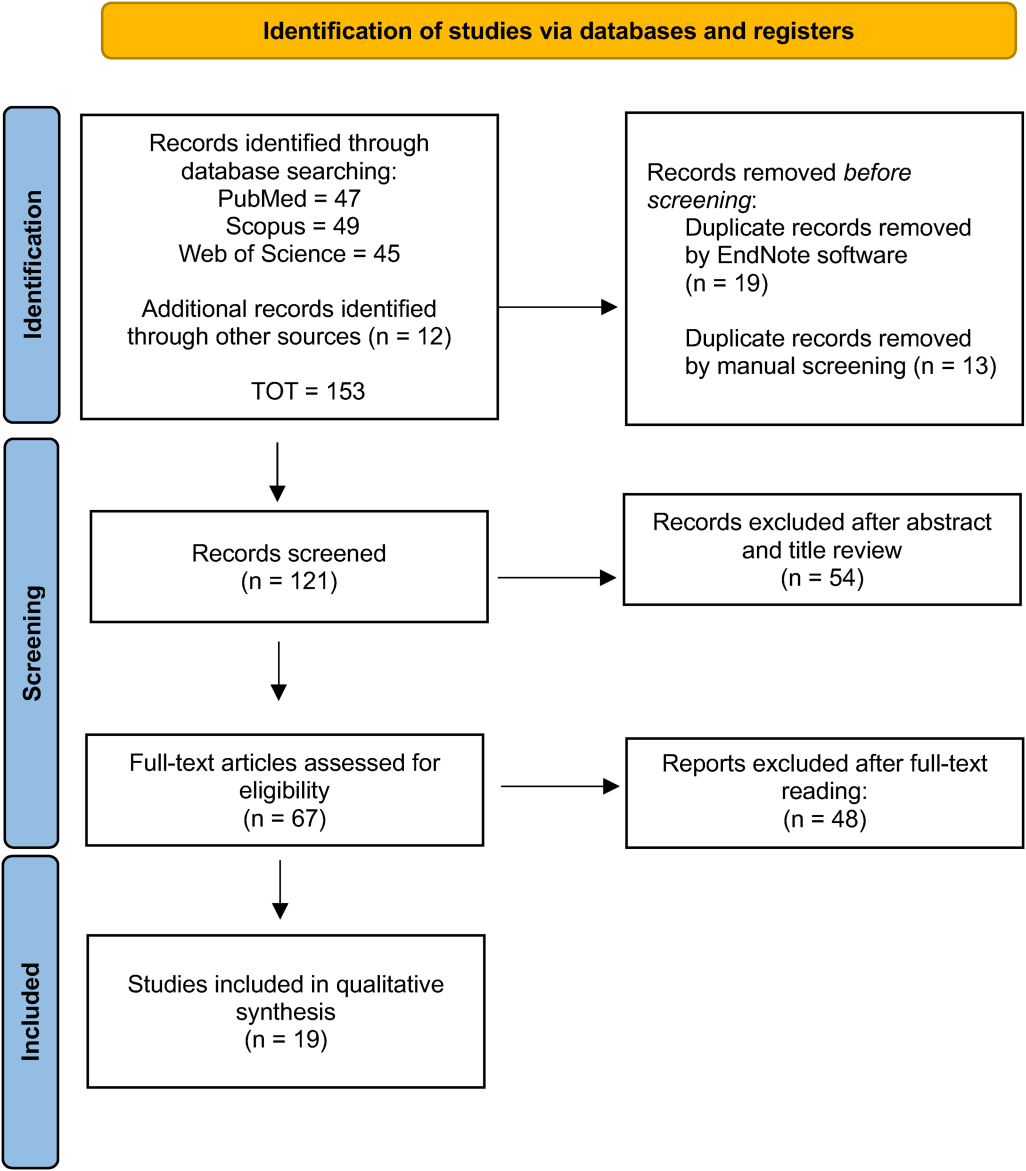
PRISMA flowchart.

The initial search strategy identified 152 records, of which 32 were removed as they were duplicates. The screened process of 120 studies was performed based on the title and abstract, and 54 records were excluded. Subsequently, a full-text evaluation of 67 studies was carried out. Finally, based on the inclusion criteria 48 records were excluded, and 19 papers were included in the current review; a detailed flow chart of the selection process is provided in Figure 1.

### 2.6 Descriptive analysis

The selected studies were analysed to detect outcomes of interest by two authors (MC and VCAC). For each study, the following data were extracted using a pre-designed data extraction Excel sheet.

The following parameters were collected:

i. Study characteristics: the name of the first author, the year of publication, the name of the country where the study was performed, and the design of the study
ii. Main characteristics of the included patients: mean age, sex, drug, and medical history
iii. Characteristics of lip filler procedure: filler material and site of injection
iv. Characteristics of adverse reaction: type, anatomical site, treatment, and outcome

Data extraction and descriptive analysis were performed using Microsoft Excel.

### 2.7 Risk of bias assessment

Included studies underwent quality check and risk of bias assessment. This qualitative analysis was performed according to Murad's quality checklist of case series and case report (18, 19). As reported, the scale consists of four parameters, to evaluate the patient selection, exposure ascertainment, causality and reporting. Each requested field was considered as adequate, inadequate or not evaluable.

## 3 Results

### 3.1 Characteristics of included studies

Of the 67 total records assessed for eligibility, 19 were selected. The main characteristics of the included studies are reported in Table 1. All the included articles were observational studies published between 2003 and 2023. In detail, 4 studies were case series (22, 25, 29, 35) and 15 were case reports (20, 21, 23, 24, 26–28, 30–34, 36–38). Of the 19 studies, 3 were from the USA (23, 24, 29), 2 were from Brazil (28, 32), 2 were from Spain (20, 25), 2 were from Germany (21, 27), 1 was from the Netherlands (22), 1 was from Greece (26), 1 was from Korea (30), 1 was from Turkey (31), 1 was from Canada (33), 2 were from Italy (34, 38), 1 was from Iran (35), 1 was from Poland (36) and 1 was from the UK (37), as reported in Table 1.

**Table 1:** Main characteristics of the included studies.

*N*	Author, Year	Country	N. of case	Age	Sex	Filler	ADR type	Site	Onset (time after injection, months)
1	Fernandez Acenero M. J., 2003 (20)	Spain	1	48	F	Hyaluronic acid	Granulomatous foreign body reaction	Upper lip	2
2	Schmidt-Westhausen A.M., 2004 (21)	Germany	1	56	F	Silicone	Abscess	Lower lip	12
3	Dijkema J.S., 2005 (22)	Netherlands	1^a^	64	F	Poly-L-lactic acid	Granulomatous foreign body reaction	Upper lip	14
4	Leonhardt J.M., 2005 (23)	USA	1	52	F	Hyaluronic acid	Angioedema	Upper lip	0
5	Edwards P.C., 2006 (24)	USA	1	74	F	Hyaluronic acid	Granulomatous foreign body reaction	Lower lip	6
6	Sanchis-Bielsa J.M., 2009 (25)	Spain	9^b^	63	F	Silicone	Granulomatous foreign body reaction	Perioral and buccal mucosa	168
				70	F	Collagen			24
				55	F	Silicone			108
				37	M	Polyalkylimide			84
				54	F	Silicone			120
				41	F	Collagen			60
				69	F	Silicone			120
				53	F	Hyaluronic acid			48
				40		Silicone			n.d.
7	Dionyssopoulos A., 2007 (26)	Greece	1	45	F	Poly-L-lactic acid	Granulomatous foreign body reaction	Both lips	18
8	Weyand B., 2008 (27)	Germany	1	62	F	Mixed (Hyaloronic Acid + Hydroxyethyl Methacrylate + Ethyl Methacrylate)	Granulomatous foreign body reaction and pseudomonas superinfection	Both lips	n.d.
9	da Costa Miguel M.C., 2009 (28)	Brazil	1	56	F	Polymethylmethacrylate immersed in a solution of collagen	Granulomatous foreign body reaction	Right anterior inferior alveolar mucosa	12
10	Shahrabi Farahani S., 2012 (29)	USA	3	55; 57; 56.	F	Hyaluronic acid	Granulomatous foreign body reaction	Upper lip Lower lip Lower lip.	4; 24; n.d.
11	Lee S.C., 2013 (30)	Korea	1	50	F	Polymethylmethacrylate immersed in a solution of collagen	Granulomatous foreign body reaction	Upper lip	n.d.
12	Bulam H., 2015 (31)	Turkey	1	27	F	Hyaluronic acid	Angioedema	Both lips	0 (at the same time)
13	Curi M.M., 2015 (32)	Brazil	1	65	F	Hyaluronic acid	Granulomatous foreign body reaction	Upper lip	144
14	Alghonaim Y.A., 2016 (33)	Canada	1	52	F	Hyaluronic acid	Granulomatous foreign body reaction	Lower lip	1
15	Paolino G., 2017 (34)	Italy	1	41	F	Hyaluronic acid	Melanoses	Lower lip	n.d.
16	Ehsani A.H., 2019 (35)	Iran	2	25; 28	F	vitamin E	Lipogranuloma	Both lips	1
17	Kaczorowski M., 2020 (36)	Poland	1	52	F	Hyaluronic acid	Granulomatous foreign body reaction	Buccal mucosa	24
18	Wege J, 2021 (37)	UK	1	27	F	Hyaluronic acid	Lymphangioma	Upper lip	12
19	Scarano A., 2023 (38)	Italy	1	53	F	Hyaluronic acid	Granulomatous foreign body reaction	Lower lip	120

^a^ Study included 2 cases, but only 1 localized on the lips.

^b^ Study included 15 cases, but only 9 localized on the lips.

In total, 30 patients affected by lip ADRs were analyzed, of which 29 were females and only 1 was male (96.7% and 3.3%, respectively) with a mean age of 50.9 ± 12.8 years, ranging from 25 to 74 years. Comorbidities were not reported in most of the studies. Only two studies reported systemic disease of 2 patients: one affected by Hashimoto's thyroiditis and one by breast cancer (27, 36).

Hyaluronic Acid was the most commonly dermal filler used (14/30, 46.7%) (20, 23, 24, 29, 31–34, 36–38), followed by silicone (6/30, 20%) (21, 25), Poly-L-lactic acid (PLLA) (2/30, 6.7%) (22, 26), Polymethylmethacrylate (PMMA) immersed in a solution of collagen (2/30, 6.7%) (28, 30), vitamin E (2/30, 6.7%) (35), collagen (2/30, 6.7%) (25), Polyalkylimide (1/30, 3.3%), and mixed filler, composed by HA, Hydroxyethyl Methacrylate and Ethyl Methacrylate (1/30, 3.3%) (27).

Regarding reporting ADRs, no study specified whether it was reported to regulatory authorities.

The upper lip was affected in 7 cases (7/30, 23.3%) (20, 22, 23, 29, 30, 32, 37), the lower lip was affected in 7 cases (7/30, 23.3%) (21, 24, 29, 33, 34, 38), and both lips were affected in 5 cases (5/30, 16.7%) (26, 27, 31, 35), followed by perioral and buccal mucosa (9/30, 30%) (25), buccal mucosa (1/30, 3.3%) (36) and alveolar mucosa (1/30, 3.3%) (28).

Most of patients underwent a biopsy and consecutively a histological evaluation to confirm the ADR, except few cases in which no biopsy was performed or it was not specified (23, 25, 26, 31).

The most common adverse filler lip reaction was granulomatous foreign body reaction (23/30, 76.6%) (20, 22, 24, 25, 27–29, 32, 33, 36, 38) (26, 30), followed by angioedema (2/30, 6.7%) (23, 31), lipogranuloma (2/30, 6.7%) (35), melanoses (1/30, 3.3%) (34), abscess (1/30, 3.3%) (21), and lymphangioma (1/30, 3.3%) (37). Of the patients who developed granulomatous foreign body reactions, one also developed superinfection by *Pseudomonas* (27).

Based on the available data, the mean time between filler lip injection and granulomatous foreign body reaction onset was 57.9 ± 54 months (median 24 months).

In detail, the mean time between filler lip injection and granulomatous foreign body reaction onset in patients who underwent filler lip with HA vs. other dermal filler types (e.g., PLLA, silicone, collagen, PMMA) was 41.4 ± 50.8 months (median 24 months, range 1–144) vs. 72.8 ± 52.5 months (median 72 months, range 12–168), respectively.

Regarding the onset time of the other types of ADRs, angioedema developed a few minutes after the HA injection (23, 31), lipogranuloma after 1 month and abscess and lymphangioma after 12 months (21, 26, 30, 35, 37).

### 3.2 Risk of bias assessment

Results from quality and risk of bias assessment are summarized in Table 2. Briefly, eleven studies fulfilled the quality checklist (20–22, 24, 28, 32, 33, 35–38). Some studies failed in “Was the outcome adequately ascertained?” since no biopsy was performed or it was not specified (23, 25, 26, 31). For the domain of causality, all studies resulted not evaluable as since the topic is adverse reactions to lip filler injections, it was not possible to evaluate causality with cessation of the drug and its reintroduction. A complete report of the quality checklist is reported in Table 2.

**Table 2:** Risk of bias assessment.

Author	Country	Domains	Selection	Ascertainment (1) (2)		Casuality	Reporting
Fernandez Acenero et al.	Spain	Ascertained	Ascertained	Ascertained	Ascertained	Not evaluable	Ascertained
Schmidt-Westhausen et al.	Germany	Ascertained	Ascertained	Ascertained	Ascertained	Not evaluable	Ascertained
Dijkema et al.	Netherlands	Ascertained	Ascertained	Ascertained	Ascertained	Not evaluable	Ascertained
Leonhardt et al.	USA	Ascertained	Ascertained	Ascertained	Unclear	Not evaluable	Ascertained
Edwards et al.	USA	Ascertained	Ascertained	Ascertained	Ascertained	Not evaluable	Ascertained
Sanchis-Bielsa et al.	Spain	Ascertained	Unclear	Ascertained	Unclear	Not evaluable	Unclear
Dionyssopoulos et al.	Greece	Ascertained	Ascertained	Ascertained	Unclear	Not evaluable	Ascertained
Weyand et al.	Germany	Ascertained	Ascertained	Ascertained	Ascertained	Not evaluable	Unclear
da Costa Miguel et al.	Brazil	Ascertained	Ascertained	Ascertained	Ascertained	Not evaluable	Ascertained
Shahrabi Farahani et al.	USA	Ascertained	Ascertained	Ascertained	Ascertained	Not evaluable	Unclear
Lee et al.	Korea	Ascertained	Ascertained	Ascertained	Ascertained	Not evaluable	Unclear
Bulam et al.	Turkey	Ascertained	Ascertained	Ascertained	Unclear	Not evaluable	Ascertained
Curi et al.	Brazil	Ascertained	Ascertained	Ascertained	Ascertained	Not evaluable	Ascertained
Alghonaim et al.	Canada	Ascertained	Ascertained	Ascertained	Ascertained	Not evaluable	Ascertained
Paolino et al.	Italy	Ascertained	Ascertained	Ascertained	Ascertained	Not evaluable	Unclear
Ehsani et al.	Iran	Ascertained	Ascertained	Ascertained	Ascertained	Not evaluable	Ascertained
Kaczorowski et al.	Poland	Ascertained	Ascertained	Ascertained	Ascertained	Not evaluable	Ascertained
Wege et al.	UK	Ascertained	Ascertained	Ascertained	Ascertained	Not evaluable	Ascertained
Scarano et al.	Italy	Ascertained	Ascertained	Ascertained	Ascertained	Not evaluable	Ascertained

## 4 Discussion

During the 21st century, interest in aesthetic treatments, including lip fillers, has significantly increased. According to ISAPS data, in 2022, 9,221,419 botulinum toxin procedures, and 4,312,037 HA filler procedures have been performed worldwide (39).

Despite the high frequency of lips augmentation procedures, few studies investigated adverse reactions to filler lips (11, 40). In recent years, few studies have analyzed adverse reactions to dermal filler injections in the head and neck area (41). A meta-analysis performed in 2021 investigated adverse reactions following HA injections for lip augmentation, including 10 records for quantitative synthesis (40).

Nevertheless, to the best of our knowledge, this is the first study that analyzes the ADRs associated with all types of dermal fillers after lips augmentation.

Regarding the worldwide incidence, in our review, most adverse reactions after filler lips injection were reported in the USA, followed by Brazil. So, our results are consistent with that USA and Brazil ranked first and second for the number of aesthetic procedures performed in 2022, according to the ISAPS (39). Recently, Italy has also made significant advancements in this field. In 2023, the use of dermal fillers was extended to include dentists, aiming to enhance the psychosocial well-being of patients, following the amendment of law no. 409/1985 (42). According to a survey by the Italian Society of Aesthetic Medicine, Italians are the most frequent users of dermal fillers, second only to Americans, being mostly women (42).

The present review highlighted the predominance of female patients undergoing aesthetic treatments, specifically filler injections, compared to males. Furthermore, the average age of patients who underwent lip filler injections was 50.9 ± 12.8 years. This gender difference emerging from our study is consistent with a study performed by Dunaev JL et al., which investigated cosmetic practice attitudes among midlife women (43). According to this study, as women approach middle age, they become statistically more likely to pursue cosmetic surgery, probably due to insecurities associated with aging and fear of negative appearance evaluation (43). In addition to a greater adherence by women to lip filler procedures, the female prevalence in the present study could also be attributable to that women are involved in more ADR reports than men across different countries, although in some cases, men experience more serious ADRs (44).

According to the study performed by Brabete AC et al., differences in the number of reported ADRs can be linked to sex-related factors, including hormones, genetics, metabolic processes, anatomical characteristics, and organ function. For example, generally, females weigh less than males; however, few drugs are administered based on weight, and if/when there is a “one size fits all” dose, the result will be a higher exposure among women (44).

Immunity response is influenced by multiple factors including gender and sex hormones. Males and females differ in their immunological responses to foreign and self-antigens and show distinctions in innate and adaptive immune responses. Generally, females have stronger cellular and humoral immune reactions compared with males (45, 46).

Based on biological and chemical characteristics there are several types of dermal fillers. The ideal injectable product is highly biocompatible, easily injectable thanks to its favorable rheology, and produces an acceptably long-lasting effect (47). Based on their duration, fillers are classified as absorbable and permanent. Absorbable fillers are temporary, biodegradable, and last less than 1 year (e.g., HA, collagen). Permanent fillers are on-absorbable and non-biodegradable (e.g., PMMA, Silicone) (48).

The biocompatibility and the ability to reverse the effects of injection using the intralesional hyaluronidase enzyme made HA the most commonly used dermal filler (49). In line with what was previously reported, also in the present study, HA was the most common dermal filler employed. Due to its natural origin, it is thought that HA does not have severe or persistent side effects. However, recent evidence showed that major, adverse effects may appear associated with its use, including immediate and delayed (50). In a recent study, the authors suggested not underestimating the effects of HA, as delayed and recurrent chronic inflammatory and granulomatous reactions may complicate HA fillers (50).

Based on the onset time of adverse effects, Abduljabbar H.M. et al. classified ADR as early (since hours to days post-procedure) and delayed (since weeks to years post-procedure). Among the early adverse effects of HA injection, Abduljabbar H.M. et al. reported injection site reactions (e.g., edema, pain, erythema, itching, and ecchymosis), hypersensitivity reactions, infection (e.g., Herpes simplex virus, abscess/cellulitis and Mycobacterial infection), surface irregularities and nodules, vascular occlusion; while the most common delayed adverse effect was granulomatous foreign body reaction (51).

In the present study, the most common, although rare, early filler lip reaction was angioedema and the most common delayed reaction was foreign body granulomatous reaction.

Among the ADRs associated with filler lips observed, angioedema, although rare, is the most dangerous to the patient's life as it can obstruct the upper airways (52). Regarding the onset time, in the present study, angioedema developed a few minutes after the HA injection (23, 31). It represents a hypersensitivity reaction, predominantly triggered by drug-induced mechanisms mediated by IgE (53). It manifests as a localized edematous swelling of the cutaneous or mucosal tissues, induced by a temporary increase in vascular permeability mediated by vasoactive mediators. A study repored a hypersensitivity reaction following HA injection, characterized by swelling, erythema, and induration at the injection site, occasionally accompanied by edema in the adjacent tissue, with a median duration of 15 days (51). Management of angioedema typically involves systemic corticosteroid therapy (23).

Granulomatous foreign body reaction body represents a chronic inflammatory reaction triggered by the immune system's inability to enzymatically degrade or phagocytose the foreign material (54). Despite purification processes, HA fillers may contain trace amounts of protein contaminants, representing a potential risk for hypersensitivity reactions and granuloma formation (55). The diagnosis of foreign body reactions can be difficult since the patients present with non-specific symptoms such as pain and/or swelling (56). In a study performed by Cavallieri F. et al., the authors proposed a specific nomenclature: persistent intermittent delayed edema (PIDS) to group late HA adverse reactions, characterized by late local intermittent edema, triggered by specific factors, that persists while there is HA in the tissue (57). Among the triggered specific factors, they reported systemic infections or a dental procedure before the appearance of the PIDS (57).

The incidence of granulomatous foreign body reaction following HA filler injections has been reported to range from 0.02% to 0.4% (58).

Also, according to our systematic review, a study performed by Machado RA et al. reported that the most common adverse reaction to the injection of face and neck aesthetic filling materials was granulomatous foreign body reaction in 87.1% of the patients, followed by lipogranuloma, xanthelasma-like reaction, and fibrotic reaction. Furthermore, also in this study, the most commonly used materials were silicone fillers, followed by HA (41). While in a study performed by Czumbel LM et al., the most frequent adverse effect after HA injection for lip augmentation was tenderness (88.8%), injection site swelling (74.3%), and bruising (39.5%), followed by granulomatous foreign body reaction (0.6%), herpes labialis (0.6%) and angioedema (0.3%) (40).

Regarding the onset time, although in the literature it has been reported that filler-related foreign body granulomas generally occur 6–24 months after filler injections (58); in the present study the mean time between filler lip injection and granulomatous foreign body reaction onset was 41.4 ± 50.8 months (median 24 months) in patients who underwent filler lip procedure with HA and 72.8 ± 52.5 months (median 72 months) in patients who underwent filler lip procedure with other dermal filler types (e.g., PLLA, silicone, collagen, PMMA). So, the present study highlights the importance of a follow-up for at least two years following dermal filler injection given the possibility of delayed ADR onset.

In line with the present systematic review, other studies reported delayed onset of ADRs secondary to the dermal filler injection, including the foreign body-related chronic inflammatory reaction (9).

Theoretically, the risks for immune-mediated reactions are minimized using biomaterials with improved physical characteristics (59). In practice, there is an increasing number of reports of immediate and delayed ADRs associated with dermal fillers. The reported immediate ADRs associated with filler lips are often characterized by severe injection site erythema, edema, and induration (9).

The slow onset of the foreign body-related chronic inflammatory reaction and its dynamic profile (beginning with an acute inflammatory attack and transitioning to a long-term fibrotic response) can make it difficult to predict and recognize it (60).

Duker et al. reported that patient's quality of life can be moderately to severely impacted due to the ADRs that occurred secondary to dermal fillers and the impact is greater with patients who had a non-biodegradable agent injected compared to those who had a biodegradable agent injected (61).

Given the increasing use of various dermal filler products globally, not always FDA-approved, and the wide range of ADRs associated with them it is crucial to provide a dedicated training program for healthcare professionals who use them (62). Compared to early-onset ADRs, which are localized in the injection site and resolve spontaneously; delayed ADRs are persistent and require surgical interventions for their resolution. So, it is critical to inform patients of the potential for ADR secondary to lip fillers, even months or years after the injection, and the need for ongoing follow-up visits even after the initial satisfactory result (62).

In the Authors’ opinion, just as informed consent is obtained before performing a surgical procedure (e.g., tooth extraction or dental implant surgery), healthcare professionals should also take the time to explain and inform the patient of the potential benefits and risks associated with filler lip procedures, improving patients’ awareness about the possibility of ADR onset (63).

According to the study by Edwards PC et al., the number and type of ADRs associated with filler lips reported in the literature are not completely accurate, as the total number of cases may be underestimated since often it is not reported by clinicians (9). It is noteworthy that no study included in our literature review reported anything about the appropriateness of reporting of ADRs to the pharmacovigilance office.

Thanks to their minimal invasiveness and affordable prices, filler lips procedure has become fashionable among people of all ages and socioeconomic backgrounds (64). As the use of fillers becomes increasingly more common and the skill level of those injecting is so varied, adverse events can be expected to increase as well (65). Cosmetic treatments require the same case evaluation as any other medical or elective treatment. Practitioners should gather the patient's medical and surgical history, current medications, allergies, and details of any previous cosmetic procedures (66).

Patients should be made aware of the potential risks and benefits of cosmetic procedures, including filler lips, as well as the importance of follow-up visits (43). The American Academy of Facial Esthetics has proposed a specific informed consent form to provide written information about the risks, benefits, and alternatives of the aforementioned procedure.

Since ADRs can occur, them early detection and prompt treatment may eliminate or minimize sequelae. Therefore, healthcare providers should remain vigilant in monitoring patients for potential ADRs and report any suspected cases to drug regulatory authorities (for Italy, AIFA-Italian Medicines Agency) (15). Reporting ADRs, of course, is crucial for improving patients’ safety and gaining a better understanding of the safety profiles of the drug and medical devices. In the case of suspicion of ADR associated with the use of dermal fillers, it may be more complicated to establish the causal link as it is not possible to carry out the challenge-dechallenge-rechallenge.

The present study possesses some limitations. First, the included studies are heterogeneous in terms of the dermal filler used. Moreover, not all studies reported a diagnosis confirmed by oral biopsy; for this reason, they were considered at high risk of bias. Second, some ADR might be correlated with the injection technique (operator-dependent). The absence of a standard protocol for filler lips has made it challenging to evaluate the appropriateness of the procedure. Third, in the included studies the specialty of the operator who performed the filler procedure is not specified (e.g., plastic surgery, dentistry). Moreover, although the most common, the ADRs secondary to HA could be underestimated due to the reversible effects through the use of hyaluronidase Lastly, this systematic review analyzed ADRs associated with lip fillers exclusively. However, given the increasing prevalence of dentist-led facial aesthetic treatments in some countries, it would be interesting for future studies to investigate this most critical and emergent problem.

## 5 Conclusion

Our results suggest that the development of adverse reactions can occur even after a considerable time from the aesthetic procedure mostly used by women. For this reason, it is crucial to continue both short-term and long-term follow-ups and to report ADR in the national pharmacovigilance systems, since, despite these procedures being performed with biocompatible materials, they are not devoid of ADR. Moreover, the prompt identification and reporting of ADRs to drug regulatory authorities are essential for patients’ safety.

## Data Availability

The raw data supporting the conclusions of this article will be made available by the authors, without undue reservation.

## References

[1] Board WHOE. Handbook of Resolutions and Decisions of the World Health Assembly and the Executive Board: V. 1, 1948–1972. Geneva, Switzerland: World Health Organization (1973).

[2] Baldo P Francescon S Fornasier G. Pharmacovigilance workflow in Europe and Italy and pharmacovigilance terminology. Int J Clin Pharm. (2018) 40(4):748–53. 10.1007/s11096-018-0711-z 30094557 PMC6132975

[3] Kaufman G. Adverse drug reactions: classification, susceptibility and reporting. Nurs Stand. (2016) 30(50):53–63. 10.7748/ns.2016.e10214 27507394

[4] World Health Organization. WHO Policy Perspectives on Medicines. Promoting Rational use of Medicines: Core Components. Geneva, Switzerland: World Health Organization (2002). p. 1.

[5] Guo J Fang W Wang F. Injectable fillers: current status, physicochemical properties, function mechanism, and perspectives. RSC Adv. (2023) 13(34):23841–58. 10.1039/D3RA04321E 37577103 PMC10413051

[6] Singh K Nooreyezdan S. Nonvascular complications of injectable fillers-prevention and management. Indian J Plast Surg. (2020) 53(3):335–43. 10.1055/s-0040-1721872 33500603 PMC7822713

[7] Zielke H Wolber L Wiest L Rzany B. Risk profiles of different injectable fillers: results from the injectable filler safety study (IFS study). Dermatol Surg. (2008) 34(3):326–35. discussion 35. 10.1111/j.1524-4725.2007.34066.x 18177399

[8] Luebberding S Alexiades-Armenakas M. Safety of dermal fillers. J Drugs Dermatol. (2012) 11(9):1053–8. 23135647

[9] Edwards PC Fantasia JE. Review of long-term adverse effects associated with the use of chemically-modified animal and nonanimal source hyaluronic acid dermal fillers. Clin Interv Aging. (2007) 2(4):509–19. 10.2147/cia.s382 18225451 PMC2686337

[10] Bitterman-Deutsch O Kogan L Nasser F. Delayed immune mediated adverse effects to hyaluronic acid fillers: report of five cases and review of the literature. Dermatol Rep. (2015) 7(1):5851. 10.4081/dr.2015.5851 25918619 PMC4387334

[11] Sanchez-Carpintero I Candelas D Ruiz-Rodriguez R. Dermal fillers: types, indications, and complications. Actas Dermosifiliogr. (2010) 101(5):381–93. 10.1016/j.ad.2010.01.004 20525480

[12] Diwan Z Trikha S Etemad-Shahidi S Parrish N Rennie C. Evaluation of current literature on complications secondary to lip augmentation following dermal filler injection. J Clin Aesthet Dermatol. (2023) 16(7):26–33. 37560504 PMC10409513

[13] Kroumpouzos G Treacy P. Hyaluronidase for dermal filler complications: review of applications and dosage recommendations. JMIR Dermatol. (2024) 7:e50403. 10.2196/50403 38231537 PMC10836581

[14] Mwakawanga DL Kilonzi M Philipo EG Martine A Mbilinyi T Kileo NF Pharmacovigilance and adverse drug reactions reporting: healthcare Providers’ experiences from southern highland Tanzania. Adv Pharmacol Pharm Sci. (2023) 2023:5537592. 10.1155/2023/5537592 37876921 PMC10593552

[15] La Mantia G Buttacavoli F Panzarella V Colella G Capuano A Sportiello L Oro-Dental pharmacovigilance in the digital age: promoting knowledge, awareness, and practice in Italy through a smart combined system—a conference at the 30th national congress of the Italian college of university professors of dental disciplines. Oral. (2023) 3(3):411–9. 10.3390/oral3030033

[16] Al Mashhrawi YM AlNojaidi TF Alkhaldi RA Alshami NS Alhadlaq AS. Awareness and knowledge of the adverse effects of dermal fillers among the Saudi population: a cross-sectional study. Cureus. (2023) 15(6):e40322. 10.7759/cureus.40322 37448388 PMC10337987

[17] Page MJ McKenzie JE Bossuyt PM Boutron I Hoffmann TC Mulrow CD The PRISMA 2020 statement: an updated guideline for reporting systematic reviews. Br Med J. (2021) 372:n71. 10.1136/bmj.n71 33782057 PMC8005924

[18] Pannone G Caponio VCA De Stefano IS Ramunno MA Meccariello M Agostinone A Lung histopathological findings in COVID-19 disease - a systematic review. Infect Agent Cancer. (2021) 16(1):34. 10.1186/s13027-021-00369-0 34001199 PMC8127295

[19] Murad MH Sultan S Haffar S Bazerbachi F. Methodological quality and synthesis of case series and case reports. BMJ Evid Based Med. (2018) 23(2):60–3. 10.1136/bmjebm-2017-110853 29420178 PMC6234235

[20] Fernandez-Acenero MJ Zamora E Borbujo J. Granulomatous foreign body reaction against hyaluronic acid: report of a case after lip augmentation. Dermatol Surg. (2003) 29(12):1225–6. 10.1097/00042728-200312000-00018 14725668

[21] Schmidt-Westhausen AM Frege J Reichart PA. Abscess formation after lip augmentation with silicone: case report. Int J Oral Maxillofac Surg. (2004) 33(2):198–200. 10.1054/ijom.2002.0467 15050078

[22] Dijkema SJ van der Lei B Kibbelaar RE. New-fill injections may induce late-onset foreign body granulomatous reaction. Plast Reconstr Surg. (2005) 115(5):76e–8e. 10.1097/01.PRS.0000157022.61766.5D 15809577

[23] Leonhardt JM Lawrence N Narins RS. Angioedema acute hypersensitivity reaction to injectable hyaluronic acid. Dermatol Surg. (2005) 31(5):577–9. 10.1097/00042728-200505000-00017 15962746

[24] Edwards PC Fantasia JE Iovino R. Foreign body reaction to hyaluronic acid (restylane): an adverse outcome of lip augmentation. J Oral Maxillofac Surg. (2006) 64(8):1296–9. discussion 9. 10.1016/j.joms.2006.04.028 16860228

[25] Sanchis-Bielsa JM Bagan JV Poveda R Salvador I. Foreign body granulomatous reactions to cosmetic fillers: a clinical study of 15 cases. Oral Surg Oral Med Oral Pathol Oral Radiol Endod. (2009) 108(2):237–41. 10.1016/j.tripleo.2009.03.032 19615662

[26] Dionyssopoulos A Nikolis A Patsatsi A Sotiriadis D. Granulomas of the lips: a rare complication after injection of polylactic acid for aesthetic augmentation. J Plast Reconstr Aesthet Surg. (2007) 60(9):1079–80. 10.1016/j.bjps.2007.04.002 17532279

[27] Weyand B Menke H. Case report: adverse granulomatous reaction (granuloma formation) and pseudomonas superinfection after lip augmentation by the new filler DermaLive®. Eur J Plast Surg. (2008) 30(6):291–5. 10.1007/s00238-007-0183-1

[28] da Costa Miguel MC Nonaka CF dos Santos JN Germano AR de Souza LB. Oral foreign body granuloma: unusual presentation of a rare adverse reaction to permanent injectable cosmetic filler. Int J Oral Maxillofac Surg. (2009) 38(4):385–7. 10.1016/j.ijom.2009.01.013 19243914

[29] Farahani SS Sexton J Stone JD Quinn K Woo SB. Lip nodules caused by hyaluronic acid filler injection: report of three cases. Head Neck Pathol. (2012) 6(1):16–20. 10.1007/s12105-011-0304-9 21984020 PMC3311950

[30] Lee SC Kim JB Chin BR Kim JW Kwon TG. Inflammatory granuloma caused by injectable soft tissue filler (artecoll). J Korean Assoc Oral Maxillofac Surg. (2013) 39(4):193–6. 10.5125/jkaoms.2013.39.4.193 24471042 PMC3858123

[31] Bulam H Sezgin B Tuncer S Findikcioglu K Cenetoglu S. A severe acute hypersensitivity reaction after a hyaluronic acid with lidocaine filler injection to the lip. Arch Plast Surg. (2015) 42(2):245–7. 10.5999/aps.2015.42.2.245 25798402 PMC4366712

[32] Curi MM Cardoso CL Curra C Koga D Benini MB. Late-onset adverse reactions related to hyaluronic acid dermal filler for aesthetic soft tissue augmentation. J Craniofac Surg. (2015) 26(3):782–4. 10.1097/SCS.0000000000001358 25950527

[33] Alghonaim YA Solomon PD. Bilateral lower-lip foreign body granuloma secondary to hyaluronic acid injection. Plastic Surgery Case Studies. (2016) 2(2):16–7. 10.1177/2513826X1600200203

[34] Paolino G Garelli V Didona D Cantisani C Rossi A Donati P Melanosis of the lower lip subverted by filler injection: a simulator of early mucosal melanoma. Australas J Dermatol. (2017) 58(1):71–2. 10.1111/ajd.12475 28195323

[35] Ehsani AH Ansari MS Ghanadan A Mehdizade Rayeni N Noormohammad Poor P Ansari M. Serious complication as a result of lip augmentation with vitamin E. J Cosmet Dermatol. (2019) 18(6):1632–4. 10.1111/jocd.12926 30924223

[36] Kaczorowski M Nelke K Luczak K Halon A. Filler migration and florid granulomatous reaction to hyaluronic acid mimicking a buccal tumor. J Craniofac Surg. (2020) 31(1):e78–e9. 10.1097/SCS.0000000000005928 31634310

[37] Wege J Anabtawi M Blackwell MA Patterson A. Lymphangioma formation following hyaluronic acid injection for lip augmentation. Cureus. (2021) 13(1):e12929. 10.7759/cureus.12929 33654610 PMC7910224

[38] Scarano A Di Carmine MS Lucchina AG Giacomello M Petrini M Amore R Chronic lip edema and pain secondary to lip augmentation procedure: histological, scanning electron microscopy and x-ray microanalysis evaluation. Eur Rev Med Pharmacol Sci. (2023) 27(3 Suppl):147–52. 10.26355/eurrev_202304_31334 37129326

[39] Available online at: https://www.isaps.org/media/a0qfm4h3/isaps-global-survey_2022.pdf (accessed April 12, 2024).

[40] Czumbel LM Farkasdi S Gede N Miko A Csupor D Lukacs A Hyaluronic acid is an effective dermal filler for lip augmentation: a meta-analysis. Front Surg. (2021) 8:681028. 10.3389/fsurg.2021.681028 34422892 PMC8377277

[41] Machado RA Oliveira LQ Martelli-Junior H Pires FR Carvas JB Rogerio VE Adverse reactions to the injection of face and neck aesthetic filling materials: a systematic review. Med Oral Patol Oral Cir Bucal. (2023) 28(3):e278–e84. 10.4317/medoral.25713 36565218 PMC10181027

[42] Grippaudo C Oliva A Ricci B Grassi S Bartoletti E Grippaudo FR. The use of dermal fillers in dentistry in Italy: clinical and medico-legal aspects. Dent Cadmos. (2018) 86(04):238–52. 10.19256/d.cadmos.04.2018.06

[43] Dunaev JL Schulz JL Markey CN. Cosmetic surgery attitudes among midlife women: appearance esteem, weight esteem, and fear of negative appearance evaluation. J Health Psychol. (2018) 23(1):59–66. 10.1177/1359105316642249 27114214

[44] Brabete AC Greaves L Maximos M Huber E Li A Le ML. A sex- and gender-based analysis of adverse drug reactions: a scoping review of pharmacovigilance databases. Pharmaceuticals (Basel). (2022) 15(3):298. 10.3390/ph15030298 35337096 PMC8950058

[45] Klein SL Flanagan KL. Sex differences in immune responses. Nat Rev Immunol. (2016) 16(10):626–38. 10.1038/nri.2016.90 27546235

[46] Nussinovitch U Shoenfeld Y. The role of gender and organ specific autoimmunity. Autoimmun Rev. (2012) 11(6-7):A377–85. 10.1016/j.autrev.2011.11.001 22100310

[47] Cassuto D Bellia G Schiraldi C. An overview of soft tissue fillers for cosmetic dermatology: from filling to regenerative medicine. Clin Cosmet Investig Dermatol. (2021) 14:1857–66. 10.2147/CCID.S276676 34992400 PMC8710524

[48] Witmanowski H Blochowiak K. Another face of dermal fillers. Postepy Dermatol Alergol. (2020) 37(5):651–9. 10.5114/ada.2019.82859 33240002 PMC7675084

[49] Colon J Mirkin S Hardigan P Elias MJ Jacobs RJ. Adverse events reported from hyaluronic acid dermal filler injections to the facial region: a systematic review and meta-analysis. Cureus. (2023) 15(4):e38286. 10.7759/cureus.38286 37261136 PMC10226824

[50] Alijotas-Reig J Garcia-Gimenez V. Delayed immune-mediated adverse effects related to hyaluronic acid and acrylic hydrogel dermal fillers: clinical findings, long-term follow-up and review of the literature. J Eur Acad Dermatol Venereol. (2008) 22(2):150–61. 10.1111/j.1468-3083.2007.02354.x 18211407

[51] Abduljabbar MH Basendwh MA. Complications of hyaluronic acid fillers and their managements. J Dermatol Dermatol Surg. (2016) 20(2):100–6. 10.1016/j.jdds.2016.01.001

[52] Bouckaert M Bouckaert M Wood NH Khammissa R Lemmer J Feller L. Oral medicine case book 64: some aspects of the pathophysiology of angioedema with special reference to the upper aerodigestive tract. SADJ. (2014) 69(9):420–3. 26571926

[53] Neville BW Damm DD Allen CM Chi AC. Allergies and Immunologic Diseases. Color Atlas of Oral and Maxillofacial Diseases. St. Louis, MI: Elsevier (2019). p. 205–22.

[54] Funt D Pavicic T. Dermal fillers in aesthetics: an overview of adverse events and treatment approaches. Clin Cosmet Investig Dermatol. (2013) 6:295–316. 10.2147/CCID.S50546 24363560 PMC3865975

[55] Mamelak AJ Katz TM Goldberg LH Graves JJ Kaye VN Friedman PM. Foreign body reaction to hyaluronic acid filler injection: in search of an etiology. Dermatol Surg. (2009) 35(Suppl 2):1701–3. 10.1111/j.1524-4725.2009.01350.x 19807767

[56] Ando A Hatori M Hagiwara Y Isefuku S Itoi E. Imaging features of foreign body granuloma in the lower extremities mimicking a soft tissue neoplasm. Ups J Med Sci. (2009) 114(1):46–51. 10.1080/03009730802602455 19242872 PMC2852748

[57] Cavallieri FA Balassiano LKA Bastos JT Fontoura GHM Almeida AT. Persistent, intermittent delayed swelling (PIDS) and intermittent swelling: late adverse reactions to hyaluronic acid fillers. Surg Cosmetic Dermatol. (2017) 9(3):231–4. 10.5935/scd1984-8773.201793931

[58] Lee JM Kim YJ. Foreign body granulomas after the use of dermal fillers: pathophysiology, clinical appearance, histologic features, and treatment. Arch Plast Surg. (2015) 42(2):232–9. 10.5999/aps.2015.42.2.232 25798398 PMC4366708

[59] Cohen JL. Understanding, avoiding, and managing dermal filler complications. Dermatol Surg. (2008) 34(Suppl 1):S92–9. 10.1111/j.1524-4725.2008.34249.x 18547189

[60] Carnicer-Lombarte A Chen ST Malliaras GG Barone DG. Foreign body reaction to implanted biomaterials and its impact in nerve neuroprosthetics. Front Bioeng Biotechnol. (2021) 9:622524. 10.3389/fbioe.2021.622524 33937212 PMC8081831

[61] Duker D Erdmann R Hartmann V Nast A Rzany B Bachmann F. The impact of adverse reactions to injectable filler substances on quality of life: results from the Berlin injectable filler safety (IFS) - study. J Eur Acad Dermatol Venereol. (2016) 30(6):1013–20. 10.1111/jdv.13594 26916470

[62] Kyriazidis I Spyropoulou GA Zambacos G Tagka A Rakhorst HA Gasteratos K Adverse events associated with hyaluronic acid filler injection for non-surgical facial aesthetics: a systematic review of high level of evidence studies. Aesthetic Plast Surg. (2024) 48(4):719–41. 10.1007/s00266-023-03465-1 37563436

[63] Mauceri R Arduini S Coppini M Bazzano M Trujillo I Campisi G. Drug assumption and awareness about adverse drug reactions. The right to know. The case of the bone-modyfing agents: a systematic review. Front Oral Health. (2024) 5:1441601. 10.3389/froh.2024.1441601 39148955 PMC11324537

[64] Urdiales-Galvez F Delgado NE Figueiredo V Lajo-Plaza JV Mira M Moreno A Treatment of soft tissue filler complications: expert consensus recommendations. Aesthetic Plast Surg. (2018) 42(2):498–510. 10.1007/s00266-017-1063-0 29305643 PMC5840246

[65] Fitzgerald R Bertucci V Sykes JM Duplechain JK. Adverse reactions to injectable fillers. Facial Plast Surg. (2016) 32(5):532–55. 10.1055/s-0036-1592162 27680525

[66] Arlette JP Froese AL Singh JK. Soft tissue filler therapy and informed consent: a Canadian review. J Cutan Med Surg. (2022) 26(1):50–6. 10.1177/12034754211032542 34310242 PMC8750135

